# A Combined Experimental and Finite Element Analysis Method for the Estimation of Eddy-Current Loss in NdFeB Magnets

**DOI:** 10.3390/s140508505

**Published:** 2014-05-14

**Authors:** Radu Fratila, Abdelkader Benabou, Abdelmounaïm Tounzi, Jean-Claude Mipo

**Affiliations:** 1 L2EP/Université Lille1, 59655 Villeneuve d'Ascq, France; E-Mails: abdelkader.Benabou@univ-lille1.fr (A.B.); mounaim.tounzi@univ-lille1.fr (A.T.); 2 Valeo Equipements Electriques Moteur, 94046 Créteil Cedex, France; E-Mail: jean-claude.mipo@valeo.com

**Keywords:** eddy-current loss, iron loss, permanent magnet

## Abstract

NdFeB permanent magnets (PMs) are widely used in high performance electrical machines, but their relatively high conductivity subjects them to eddy current losses that can lead to magnetization loss. The Finite Element (FE) method is generally used to quantify the eddy current loss of PMs, but it remains quite difficult to validate the accuracy of the results with complex devices. In this paper, an experimental test device is used in order to extract the eddy current losses that are then compared with those of a 3D FE model.

## 1. Introduction

Due to their high energy density, rare earth permanent magnets are widely used in electrical machines. However, as these magnets are electrically conductive, high spatial frequencies and temporal harmonics can lead to significant eddy current loss density. In this case, irreversible demagnetization can occur because of the temperature rise [1], therefore, it is of importance to calculate with accuracy these losses. This can be achieved by solving Maxwell's equations either analytically or numerically by using the FE method [2,3]. Nevertheless, the validation of the results remains quite difficult to realize on an electrical machine.

In this paper, a dedicated test device, to measure the eddy current losses in sintered NdFeB magnets, is built. A 3D FE analysis of the eddy current losses is performed and results are compared with the experiment by a loss balance technique combining experiment and FE results.

## 2. Measurement System and Approach of Eddy Current Losses

**Figure 1.**
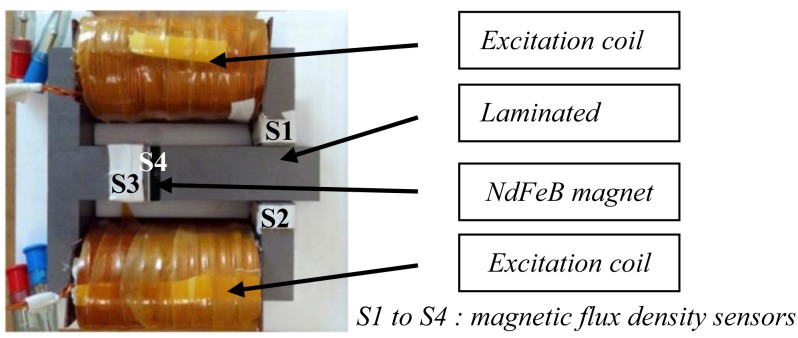
Experimental device.

The test device used for the measurements of the eddy current loss is presented in Figure 1. Its main dimensions are:

- Magnetic circuit: 130 × 140 × 60 mm (LxWxD);
- NdFeB magnet: 4 × 30 × 60 mm (LxWxD);
- Air gap: 5 mm.

The test device is composed of a magnetic circuit made of high performance electrical steel (FeSi NO 20) of 0.2 mm thickness and an iron loss density of 15 W/kg @ 1T-400 Hz. A sintered NdFeB (NEOFLUX-GSN35), with a remnant flux density of B_r_ = 1.214 T and an identified electrical conductivity of *σ* = 0.7 MS/m, is placed in the air gap of the magnetic circuit. It is subject to an alternating field created by two identical excitation coils (each with 400 turns) connected in parallel. The air-gap width, the supply frequency and the magnetic flux density can be varied.

In practice, the direct measurement of eddy current losses in the permanent magnet cannot be easily achieved. Indeed, the total losses *P_t_* are composed of the copper losses *P_Cu_* of both excitation coils, iron losses *P_iron_* of the laminated core and eddy current losses *P_PM_* in the permanent magnet as given in Equation (1). The copper losses can be deduced from direct measurements using Equation (2):

(1) $$P_{t}=P_{Cu}+P_{\text{iron}}+P_{PM}$$

(2) $$P_{Cu}=R_{p}I_{p}^{2}$$

where *R_p_* is the measured parallel resistance of the coils and *I_p_* the measured parallel current. But, the dissociation between the iron losses in the magnetic core and the eddy current losses in the PM is not straightforward. One way to achieve this goal consists in quantifying accurately the iron losses of the device by another approach in order to be able to subtract them in Equation (1). In our case, these losses are obtained from the FE calculation using the Bertotti approach [4] which is based on the decomposition of the iron losses in three contributions:

(3) $$P_{\text{iron}}=P_{h}+P_{cl.}+P_{\text{exc}}=k_{h}fB_{m}^{\alpha }+k_{cl.}f^{2}B_{m}^{2}+k_{\text{exc}}f^{1.5}B_{m}^{1.5}$$

where *P_h_*, *P_cl_* and *P_exc_* are, respectively, the quasi-static hysteresis, classic eddy currents and excess losses. The parameters *k_h_, α, k_cl_* *k_exc_* are parameters obtained from data fitting with the experiment. Then, with the copper and iron losses identified, the eddy current losses in the PM can be extracted from the following power balance:

(4) $$P_{PM}=P_{t}-P_{Cu}+P_{\text{iron}-FE}$$

## 3. Numerical Approach

The validity of the proposed approach is verified by realizing 3D Finite Element (FE) simulations with *code_Carmel*, the FE solver developed by the laboratory. The calculation of eddy currents losses in the PM is performed by solving numerically, in transient time-steps, both magneto-dynamic formulations: the electric ***A****-φ* and magnetic ***T****-Ω* formulations presented in Equations (5) and (6) in the PM domain. All calculations were done using 33 points per period, therefore the time step varies with the used frequency.

(5) $$\text{A}-\varphi :\left\{ \begin{array}{l} \text{curl}\frac{1}{\mu }\left(\text{curl}\;\text{A}-{\text{B}}_{\text{r}}\right)+\sigma \left(\frac{\partial \text{A}}{\partial t}+\text{grad}\varphi \right)={\text{J}}_{\text{S}}\\ \text{div}\sigma \left(\frac{\partial \text{A}}{\partial t}+\text{grad}\varphi \right)=0 \end{array}\right.$$

(6) $$\text{T}-\Omega :\left\{ \begin{array}{l} \text{curl}\frac{1}{\sigma }\left(\text{curl}\;\text{T}+\text{curl}\;{\text{H}}_{\text{S}}\right)+\mu \frac{\partial }{\partial t}\left(\text{T}+{\text{H}}_{\text{S}}-\text{grad}\Omega +{\text{H}}_{\text{c}}\right)=0\\ \text{div}\mu \left({\text{H}}_{\text{s}}+\text{T}-\text{grad}\Omega +{\text{H}}_{\text{c}}\right)=0 \end{array}\right.$$

where **A** is the magnetic vector potential, *φ* is the electric scalar potential, **B_r_** is the remanent magnetic flux density, **J_s_** is the current density source term that is assumed known, *σ* the electrical conductivity **T** the electric vector potential, *Ω* the magnetic scalar potential, **H_s_** the magnetic field source, **H_c_** is the coercive field and *μ* the magnetic permeability. The eddy current density **J** is then calculated according to each formulation as:

(7) $$\text{A}-\varphi :\;\text{J}=\sigma \left(-\frac{\partial \text{A}}{\partial t}-\text{grad}\varphi \right)$$

(8) T−Ω:J=curlT

The eddy currents losses *P_PM-FE_* are calculated within the permanent magnet considering a linear magnetic characteristic of the magnet and a non-linear, and non-conductive, magnetic characteristic of the laminated core. Thus, the eddy current losses in the magnet are calculated by:

(9) $$P_{PM-FE}=\frac{1}{\rho }\sum_{e=1}^{n}\frac{{\left|J_{PM}\right|}^{2}}{\sigma }[\text{W}/\text{kg}]$$

where *n* is the total number of elements of the mesh in the magnet, *ρ, σ* and *J_PM_* are, respectively, the mass density, the electrical conductivity and the eddy current density of the element *e*. Besides, the iron losses are calculated in the post-processing step of the FE calculation using the following equation applied to the laminated core domain:

(10) $$P_{\text{iron}-FE}=\sum_{e=1}^{n}\left(k_{h}f{\left(\frac{\Delta B}{2}\right)}^{\alpha }+\frac{k_{cl.}}{2\pi^{2}}\frac{1}{T}\int_{0}^{T}{\left(\frac{d\text{B}}{dt}\right)}^{2}dt+\frac{k_{\text{exc}}}{8.764}\frac{1}{T}\int_{0}^{T}{\left|\frac{d\text{B}}{dt}\right|}^{1.5}dt\right)$$

where the coefficients 2π^2^ and 8.764 are used to keep the same parameters as in Equation (3).

## 4. Measurements and Comparison with Numerical Results

**Figure 2.**
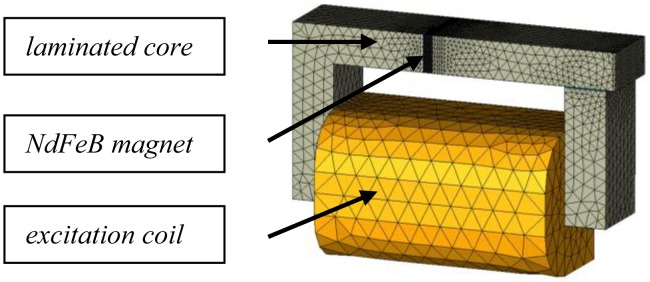
FE mesh of ¼ of the experimental device.

In Figure 2, we present the numerical model used to analyze the eddy current losses. Due to the symmetries of the device, only the quarter of the system is simulated with a mesh composed of 61,213 first order tetrahedral elements and 11,784 nodes. In order to account for the eddy currents for frequencies up to 2 kHz, the mesh of the permanent magnet is realized so that at least three elements are included in the skin depth.

**Figure 3.**
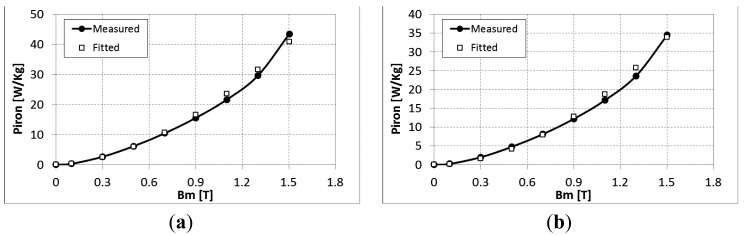
Iron losses at 400 Hz for (**a**) rolling and (**b**) transverse directions.

From measurements on an Epstein frame, the parameters of the iron loss model were identified for different frequencies (5–600 Hz) and different peak values of flux density *B_m_* (0–1.5 T). The identified parameters of the model are listed in Table 1 and Figure 3, the model behavior is presented for both directions the rolling and transverse directions of the lamination at 400 Hz.

**Figure 4.**
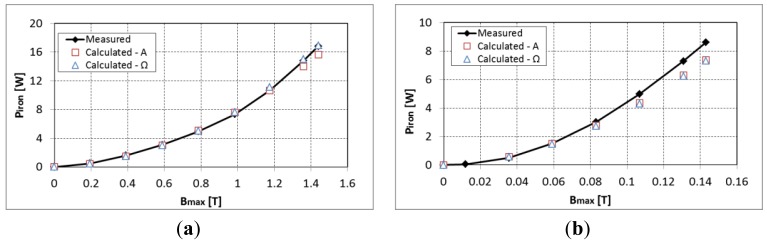
Calculation and measurement of the iron losses in the experimental device at (**a**) 50 Hz and (**b**) 600 Hz.

First, the validity of the iron loss model is verified without the permanent magnet for an air-gap of 1mm. The iron losses measured from the total losses minus the copper losses are compared to the calculated ones at 50 Hz and 600 Hz with a symmetric excitation (see Figure 4). A good approximation is observed between the measured and estimated iron losses. Giving the high inductance of the system at 600 Hz, the maximum flux density that is reached is around 0.15 T.

**Figure 5.**
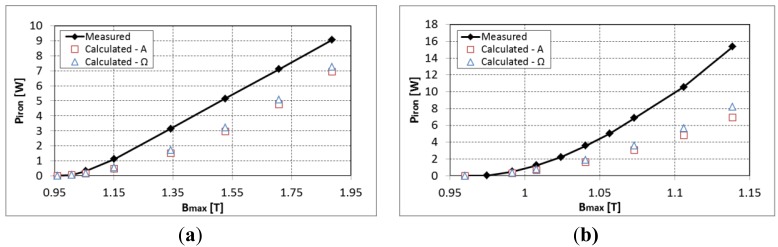
Calculation and measurement of the iron losses in the experimental device with an offset of 0.96 T at (**a**) 50 Hz and (**b**) 600 Hz.

The iron loss model gives good results when the B-H cycles of hysteresis are centered. The introduction of a PM in the system with an air-gap of 1mm induces an offset of the magnetic flux density of 0.96 T. As shown in other works, this offset will greatly increase the quasi-static hysteresis losses *P_h_* which our model cannot take it into account [5]. The effect of such offset that is imposed through a DC component with the power supply is presented in Figure 5 to emphasize its impact on the estimation of iron losses.

**Figure 6.**
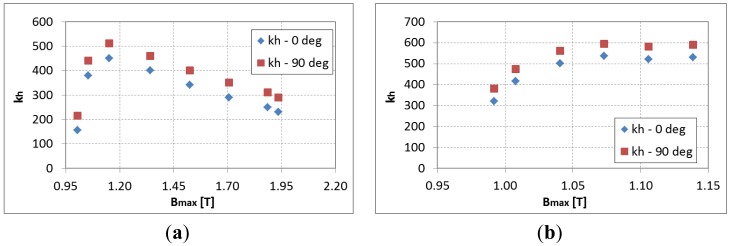
Evolution of the *k_h_* parameter at (**a**) 50 Hz and (**b**) 600 Hz with an offset of 0.96 T.

**Figure 7.**
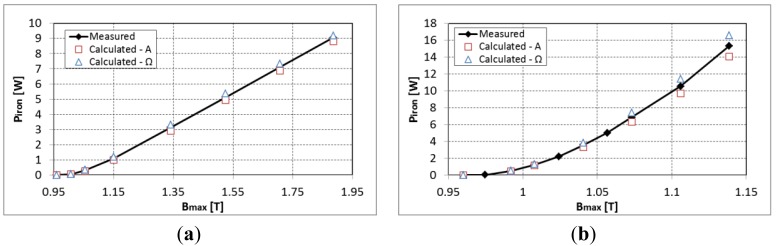
Iron losses with the new *k_h_* parameter identification at (**a**) 50 Hz and (**b**) 600 Hz (the offset is 0.96 T).

In order to compensate for this error in the computation of iron losses a polynomial variation of the *k_h_* parameter of the quasi-static hysteresis losses was identified for the considered DC magnetic flux density and depending on the *B_max_* when superposing a sinusoidal magnetic flux density. The evolution of the *k_h_* parameter is presented in Figure 6 for both directions of lamination at a frequency of 50 Hz and 600 Hz. It can be observed that initially the *k_h_* parameter increases rapidly but for important values of the peak magnetic flux density the iron losses start to decrease due to the saturation of hysteresis loop. Figure 7 shows the estimated iron losses after the re-identification of the *k_h_* parameter according to maximum magnetic flux density.

Once the iron loss model is validated for the experimental device without the magnet, the calculation of eddy currents in the permanent magnet are performed as explained previously. Table 2 shows the measurements with the sintered NdFeB magnet placed in the system. The results are presented for different frequencies and at different peak to peak levels of the magnetic flux density.

**Figure 8.**
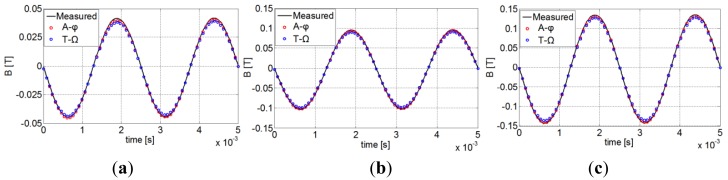
Magnetic flux density in the magnet for: (**a**) ΔB ≈ 0.1 T, (**b**) ΔB ≈ 0.2 T and (**c**) ΔB ≈ 0.3 T.

To ensure the validity of the approach, the measured global magnetic flux density of the PM is first compared with the calculated one. The obtained results for 400 Hz are presented in Figure 8 where a good approximation of the variation of the flux density in the PM is achieved.

Once the copper losses *P_Cu_* are extracted from the experiment, the iron losses *P_iron-FE_* obtained from the FE analysis are used in the power balance given by Equation (4) to deduce the eddy current losses *P_PM_* in the permanent magnet. Table 3 shows the results for a 400 Hz supply frequency.

These results are satisfactory considering the combination of experimental and modeling errors as well as the modeling hypotheses. This allows then to validate the losses obtained from the numerical model for a further use in a thermal calculation.

## 5. Conclusions

In this study an opened-circuit experimental system was fabricated to measure the eddy current losses in a NdFeB sintered magnet. A loss balance technique combining experimental and FE results was proposed in order to quantify eddy currents losses in PMs. The eddy current losses estimated with this method were then compared to those obtained directly by the 3D FEM analysis. The eddy current losses found by the 3D FE method was close to the result obtained by the proposed approach. Considering the hypothesis in the modelling procedure and experimental errors, these results confirm that the eddy current losses in a NdFeB magnet can be calculated in a satisfactory way by a 3D FE analysis.

## Figures and Tables

**Table 1.** Iron loss parameters.

	**Rolling (0 deg)**	**Transverse (90 deg)**
*k_h_*	190	251
*α*	1.841	1.623
*k_cl_.*	0.013	0.016
*k_exc_*	0	1.4E-7

**Table 2.** Loss separation from the experiment.

	**ΔB ≈ 0.1 T**				**ΔB ≈ 0.2 T**				**ΔB ≈ 0.3 T**		
f [Hz]	50	200	400	600	50	200	400	600	50	200	400
P_t_ [W]	0.34	1.06	2.71	5.31	1.77	5.41	15.8	27.3	3.48	10.4	30.7
P_Cu_ [W]	0.22	0.22	0.21	0.23	1.04	1.04	1.06	1.36	2.08	2.02	2.12
P_iron_+P_PM_ [W]	0.12	0.84	2.49	5.08	0.72	4.36	14.7	26.0	1.40	8.38	28.5

**Table 3.** Eddy current losses in the permanent magnet at 400 Hz.

			**ΔB = 0.1 T**		**ΔB = 0.2 T**		**ΔB = 0.3 T**	

			***A-φ***	***T-Ω***	***A-φ***	***T-Ω***	***A-φ***	***T-Ω***
FE calculation	P_iron-FE_	[W/kg]	0.42	0.41	1.4	1.4	2.5	2.5
	P_PM-FE_	[W/kg]	22.09	22.71	146	149	299	304

Proposed approach	P_PM_	[W/kg]	27.89	24.11	148	128	288	249
